# Bacteria Penetrate the Inner Mucus Layer before Inflammation in the Dextran Sulfate Colitis Model

**DOI:** 10.1371/journal.pone.0012238

**Published:** 2010-08-18

**Authors:** Malin E. V. Johansson, Jenny K. Gustafsson, Karolina E. Sjöberg, Joel Petersson, Lena Holm, Henrik Sjövall, Gunnar C. Hansson

**Affiliations:** 1 Department of Medical Biochemistry, University of Gothenburg, Gothenburg, Sweden; 2 Department of Internal Medicine, University of Gothenburg, Gothenburg, Sweden; 3 Department of Medical Cell Biology, Uppsala University, Uppsala, Sweden; Karolinska Institutet, Sweden

## Abstract

**Background:**

Protection of the large intestine with its enormous amount of commensal bacteria is a challenge that became easier to understand when we recently could describe that colon has an inner attached mucus layer devoid of bacteria (Johansson et al. (2008) Proc. Natl. Acad. Sci. USA 105, 15064–15069). The bacteria are thus kept at a distance from the epithelial cells and lack of this layer, as in Muc2-null mice, allow bacteria to contact the epithelium. This causes colitis and later on colon cancer, similar to the human disease Ulcerative Colitis, a disease that still lacks a pathogenetic explanation. Dextran Sulfate (DSS) in the drinking water is the most widely used animal model for experimental colitis. In this model, the inflammation is observed after 3–5 days, but early events explaining why DSS causes this has not been described.

**Principal Findings:**

When mucus formed on top of colon explant cultures were exposed to 3% DSS, the thickness of the inner mucus layer decreased and became permeable to 2 µm fluorescent beads after 15 min. Both DSS and Dextran readily penetrated the mucus, but Dextran had no effect on thickness or permeability. When DSS was given in the drinking water to mice and the colon was stained for bacteria and the Muc2 mucin, bacteria were shown to penetrate the inner mucus layer and reach the epithelial cells already within 12 hours, long before any infiltration of inflammatory cells.

**Conclusion:**

DSS thus causes quick alterations in the inner colon mucus layer that makes it permeable to bacteria. The bacteria that reach the epithelial cells probably trigger an inflammatory reaction. These observations suggest that altered properties or lack of the inner colon mucus layer may be an initial event in the development of colitis.

## Introduction

Protection of the large intestine which harbors an enormous amount (10^13^–10^14^) of commensal bacteria is a formidable challenge. To handle this we have evolved ways of maintaining a mutualistic relationship where both host and bacteria benefit. How this is managed is still an enigma, but the identification of an inner ‘firmly’ adherent mucus layer and an outer ‘loose’ non-adherent mucus layer has recently shed light on this question [1], . These two mucus layers are built around a gel-forming mucin called MUC2, a type of molecule that is preserved through evolution all the way from the early metazoans [3]. The MUC2 mucin is a highly glycosylated protein that is produced and secreted by the specialized intestinal goblet cells [4]. The human MUC2 mucin is a large molecule made of about 5,200 amino acids which is assembled into disulphide bond stabilized C-terminal dimers in the endoplasmic reticulum before translocation to the Golgi apparatus [5], [6]. After *O*-glycosylation the dimers have a mass in the range of five MDa and are then further associated into trimers via their N-terminal regions [7] to generate enormous net-like complexes [8]. After secretion, the MUC2 mucin network is hydrated and expanded in volume and forms together with other secreted proteins, a well-organized, stratified inner mucus layer [2]. This layer is dense, firmly attached to the epithelium and is insoluble in chaotropic salts. At a distance of 50 µm from the mouse epithelial cell surface, the inner attached mucus is converted into the outer mucus and expands in volume. This mucus layer is fully soluble and has its volume expanded four-times as compared to the inner adherent layer due to proteolytic cleavages [2]. The protein composition is similar in these two mucus layers as formed from a common source of secreted material. The normal bacterial flora resides in the loose mucus, whereas the inner attached mucus is impervious to bacteria and functions as a protective barrier for the epithelial cell surface [2]. This compartmentalization seems to be fundamental for the homeostasis in the highly colonized colon. The importance of the mucus barrier was further demonstrated in Muc2−/− mice where bacteria are in direct contact with the epithelial cells and are also found deep in the crypts as well as inside epithelial cells [2]. Loss of the barrier formed by the inner mucus layer triggers inflammation and development of colon cancer [2], [9], [10].

We still lack knowledge about the pathogenic mechanisms behind the inflammatory bowel disease ulcerative colitis (UC). We also lack an understanding of the mechanisms behind the colitis generated by sulfated polysaccharides. Initially, it was observed that carragenan, a sulfated galactan from seaweed, in the drinking water caused an ulcerative disease of colon in experimental animals [11]. Later it was learnt that more reproducible results were obtained by certain types of Dextran Sodium Sulfate (DSS) [12], [13]. The rodent UC model based on oral challenge with DSS has now become the most commonly used model. This compound gives wild type rodents an inflammation that starts distally after about five days and is confined to the colonic mucosa [12]–[15]. There are also a number of genetically deficient mouse models that develop colitis [16]. These include mouse strains with manipulated innate and adaptive immune systems, but still some of these models require DSS challenge [17]. Typically animals are given a 3–5% solution of DSS in their drinking water, which induces inflammation and bloody diarrhea after 4–7 days [18]. How DSS initiates the colonic inflammation is not well understood despite its wide use. We have now addressed this issue by studying the effect on the inner mucus layer secreted by mucosal explants treated with DSS, and in mice given a 3% DSS solution. We observed that DSS had a direct effect on the inner mucus layer and that this allowed bacteria to penetrate this layer before any signs of inflammation could be observed. Our observations suggest a new model for the pathogenesis of colitis where the bacterial protective properties of the inner mucus layer are in focus.

## Results

### Dextran Sodium Sulfate alters the mucus thickness and permeability in vitro

**Figure 1:**
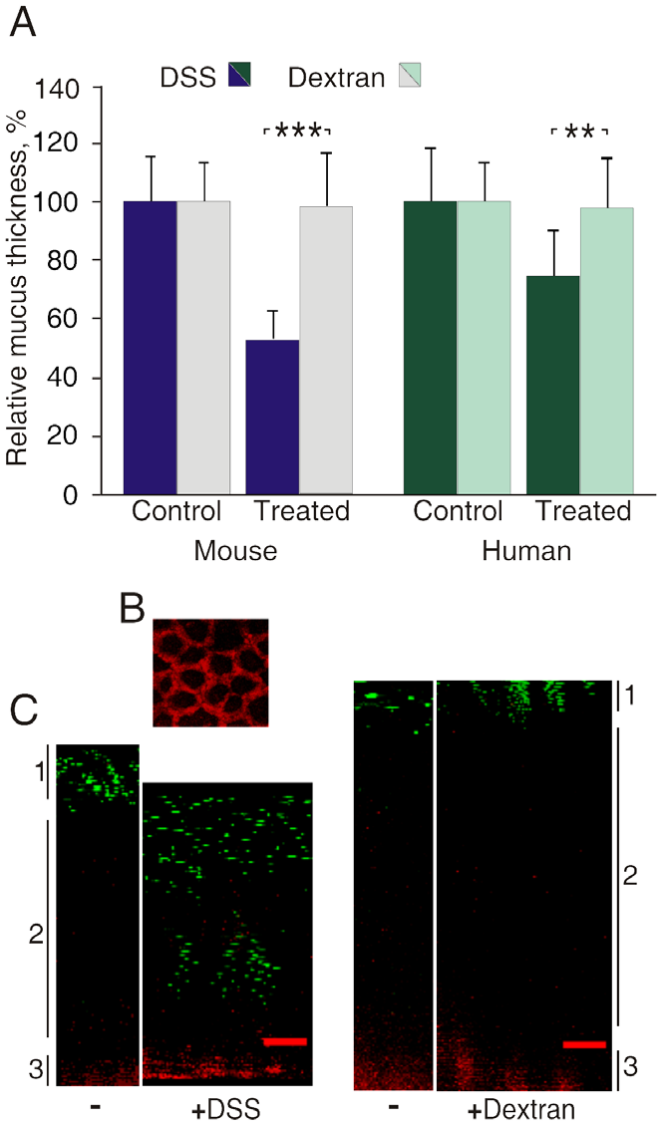
Direct effects of Dextran Sulfate (DSS) on mucus formed by explant cultures of human and mouse colon. (A) Effects of 3% DSS and 3% Dextran on the mucus thickness in mouse distal colon explants (n = 7 in each group, p<0.001) and human sigmoid colon biopsies (n = 6 in each group, p<0.01). Data is presented as mean ± SEM. The control values are normalized to 100 and the student's t-test was used to analyze the effect of the respective treatments. (B) The tissue showed a nice crypt architecture in the XY focal plane when stained with CellTracer BODIPY TR methyl ester as seen in red. (C) Z-section of an X-Y confocal image stack of secreted mucus on a mouse colon explants exposed to 3%DSS or Dextran for 15 min. The tissue was stained with a red fluorescent dye and 2 µm green fluorescent beads were left to sediment onto the mucus. During control conditions the beads remained on top of the mucus. Exposure to DSS resulted in reduced mucus thickness and beads were able to reach the epithelial surface within 15 min. Dextran had no effect on either mucus thickness or permeability. Mucus top surface (*1*), inner firm mucus (*2*), and epithelium (*3*) are marked to the left and right.

DSS is the most commonly used agent to induce colon inflammation in rodents. The mechanisms behind this effect are not clear. However, since the firmly adherent mucus layer in colon is shielding the epithelium from direct contact with bacteria and the Muc2 mucin deficient mouse lacking this mucus layer get a strong inflammation, we hypothesized that the inner mucus layer become deranged upon DSS treatment. Therefore, we first analyzed the effect of DSS on the mucus *in vitro*. In an Ussing chamber-type explant culture system, the tissue from mouse distal colon or human biopsies from sigmoid colon were mounted with the lumen upwards in a horizontal chamber with a 1.5 millimeter opening. The tissues secreted a mucus plume where the upper surface of the mucus was visualized by sparkling charcoal on its surface allowing measurement of the mucus thickness. The mucus plume was allowed to grow for 45 min, the loose mucus was removed and the thickness was measured before the apical buffer was replaced with the same buffer containing 3% DSS or 3% Dextran. The thickness of the inner firmly adherent mucus was measured again after 15 min (Fig. 1A). In explants, both from mice and humans, exposure to DSS caused a dramatic and significant decrease in the thickness of the inner mucus layer as compared to the mucus of explants treated with Dextran (Fig. 1A). The mucus thickness decreased to 53% in the mouse and to 75% in the human biopsies. The shrinking was observed already after 15 min suggesting a fast process that did not involve new mucus secretion from the epithelium. No differences in the amount of loose mucus were detected. These observations are most easily explained by a direct effect on the inner firmly adherent mucus layer itself.

To further address the effect of DSS on the mucus plume produced by explants, its permeability properties were studied by fluorescent confocal microscopy. As before, the mouse distal colon explants were allowed to secrete mucus for 45 min. The tissue was stained with a red fluorescent dye visualizing the crypt architecture nicely as an intact epithelium (Fig. 1B). The explants were analyzed by confocal XY stacks that are presented as Z-sections. First, to analyze how DSS and Dextran penetrate the mucus plume, the apical liquid was replaced with a buffer containing similarly sized FITC conjugated 3% DSS or 3% Dextran. Both these molecules penetrated the mucus layer all the way down to the epithelium within 15 min (data not shown). Secondly, green fluorescent beads with a diameter of 2 µm were allowed to sediment onto the mucus surface, and confocal XY stacks were recorded directly and after 15 min incubation with 3% DSS or 3% Dextran (Fig. 1C). The fluorescent beads were found on the top of the mucus layer in the control and Dextran treated samples. In the DSS treated explants, however, the beads penetrated the mucus and some beads were found down on the epithelial cell surface already at 15 min. This shows that DSS affects the mucus layer and allows beads, sized like most bacteria, to penetrate into the mucus. The decreased mucus thickness in the DSS treated samples was also observed as in the initial mucus measurements. DSS can thus both decrease the mucus thickness and increase the permeability of the mucus to allow particles large as bacteria to quickly penetrate the inner firmly adherent mucus of colon explants.

### No signs of inflammation with short DSS exposure

**Figure 2:**
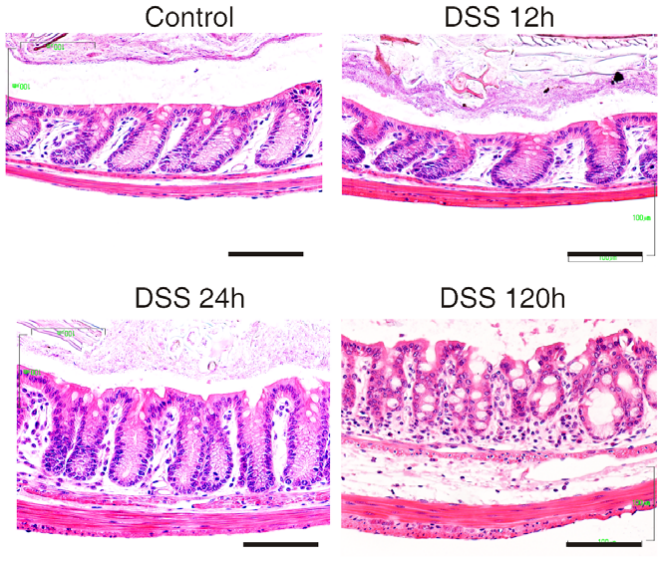
Histology of mouse colon stained with Haematoxylin/Eosin for different times of DSS exposure. No increase in the amount of infiltrating leukocytes or altered epithelial architecture as sign for inflammation was observed after 12 and 24 h of DSS treatment. Inflammation with infiltrating leukocytes and loss of normal epithelial architecture was obvious after 120 h of DSS administration. Scale bars are 100 µm.

The rapid effect of DSS on the mucus properties in the explant system suggests that DSS could have an effect on the mucus before any inflammation is observed. To analyze this, mice were given 3% DSS *ad libitum*. Sections from colon were studied, but no signs of infiltrating leukocytes or altered morphology of the epithelium could be observed within 24 h (Fig. 2). However, after 120 h a clear infiltration of leukocytes could be observed. We could thus confirm the common understanding of the DSS model that there is no inflammation during the first day of DSS treatment [14], [15], [18].

### The epithelium producing the mucus is not affected by short DSS exposure

**Figure 3:**
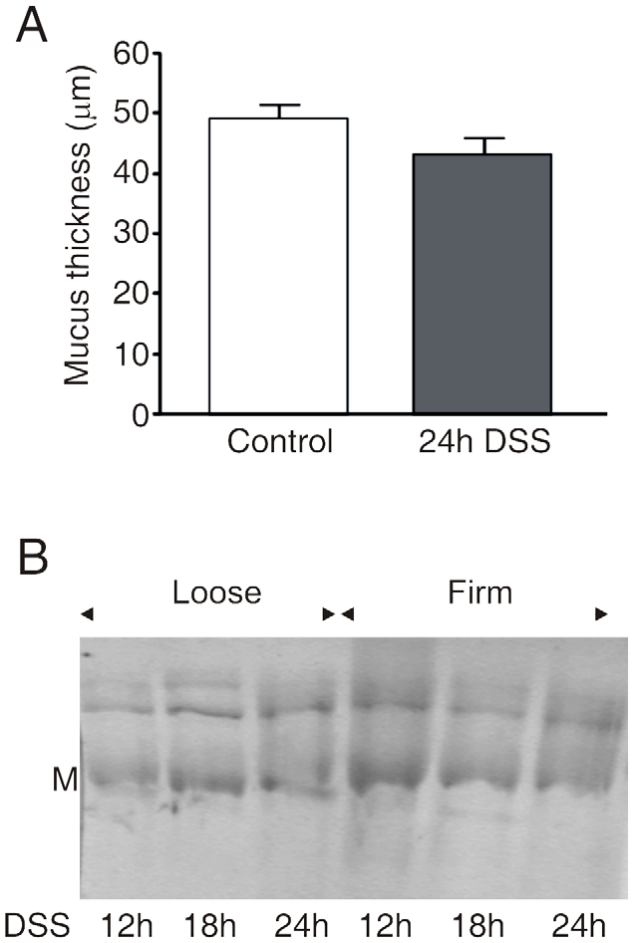
The epithelium can produce a normal mucus layer after 24 h of DSS treatment. (A) The thickness of the inner firm mucus layer was measured *in vivo* in mice after removal of the outer loose mucus layer. The measurements were made in control animals (n = 6) and in mice treated with DSS for 24 h (n = 3). No significant difference was observed. (B) The loose and firm mucus from animals treated with DSS for 12, 18 and 24 h were reduced and separated by AgPAGE and stained with Alcian blue. The Muc2 bands migrate at their normal position for a monomer (M) and non-reducible oligomers.

*In vivo* measurements of the mucus in mice have previously shown an inner firm mucus layer of about 50 µm [2]. Mice were given 3% DSS in their drinking water for 24 h and were anaesthetized. The colon was opened and a ring sealed with silicone was placed on the epithelial surface. The prepared animal was allowed a stabilization period of 1 h and then the thickness of the secreted mucus layer was measured. The mucus formed inside the ring was not subjected to DSS during this hour. This tissue had only been exposed to DSS from the drinking water during the 24 h prior to the experiment. The animals treated with DSS showed no significant alteration in the thickness of the inner firm mucus layer (Fig. 3A). This suggests that the epithelium with its mucus secreting goblet cells is functional and secretes a mucus layer of normal thickness after 24 h DSS administration.

Since the Muc2 mucin builds the mucus network [2], we analyzed the Muc2 mucin from mice that had been exposed to 3% DSS in the drinking water for 12 h, 18 h and 24 h. AgPAGE analysis revealed the normal pattern of reduced Muc2 monomer band and the larger non-reducible oligomeric bands, both in the inner firm and outer loose mucus samples (Fig. 3B). Proteomic analysis of these bands as performed previously did not reveal any alterations in the Muc2 peptide coverage or other major proteins (not shown) [2]. These experiments suggest that the epithelium is normal and that there were no major biochemical alterations in the Muc2 mucin at these early time points of DSS treatment.

### The changes in mucus properties triggered by DSS allowed bacteria to penetrate

**Figure 4:**
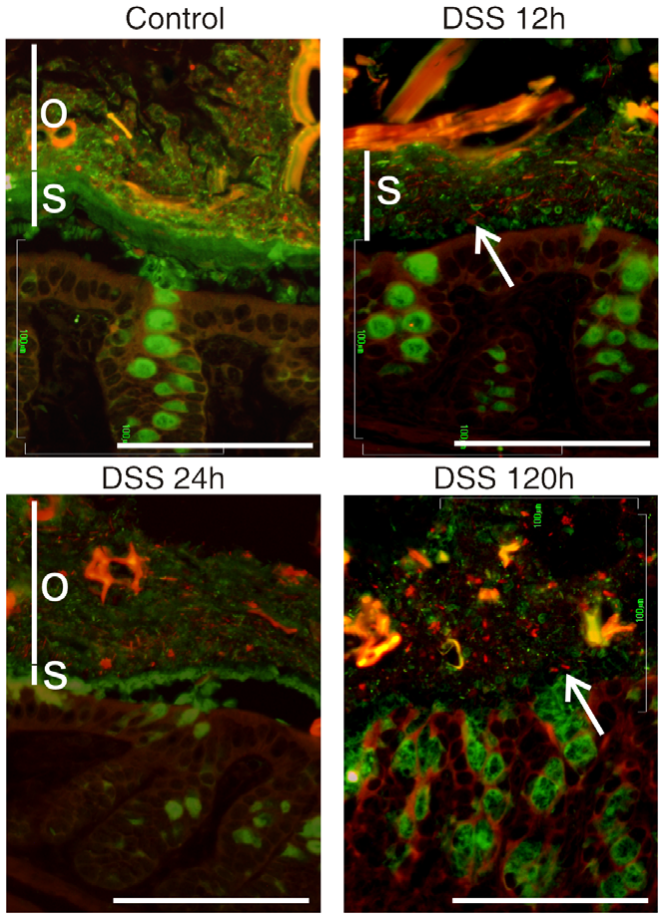
Localization of bacteria in the colon mucus of mice after DSS treatments for 12, 24 and 120 h or in nontreated control (No DSS). Bacteria were stained by fluorescent in-situ hybridization using the general bacterial rRNA probe, EUB338 conjugated with Alexa 555 (red). The mucus was visualized by immunostaining of the same section with an anti-Muc2 specific antiserum (green). Penetration of bacteria through the inner firm and stratified (*s*) mucus layer was observed already after 12 h DSS administration. The inner stratified mucus layer is marked by *s* and the outer by *o* when any of the layers could be identified. Arrows point out bacteria within the inner mucus layer (12 h) or close to the epithelium in the absence of an inner mucus layer (120 h). Scale bars are 100 µm.

As we observed that the inner mucus layer on colon explants becomes permeable to 2 µm beads already after 15 min of DSS exposure, we asked if DSS could affect the inner mucus layer prior to inflammation *in vivo*. The colon was removed from mice that had 3% DSS in their drinking water. The Carnoy fixed tissue sections were immunostainined for Muc2 (green) and by FISH (red) for bacterial 16S rRNA (Fig. 4). In non-treated control mice, the inner stratified Muc2 mucus layer (labeled *s*) was as usual observed to separate the bacteria in the outer loose mucus layer (labeled *o*) from the epithelial cells. After exposing the mice to 3% DSS in the drinking water for 12 h, the inner Muc2 layer was no longer free from bacteria and they were observed all the way down to the epithelial cell surface. Analysis of even earlier time points showed that some bacteria were able to penetrate the inner mucus layer already after 4 h. The inner mucus layer was also shown to be less well organized as the stratified lamellar organization was lost at 12 h. At 24 h the inner mucus layer had almost disappeared and at 120 h it was totally dissolved and no normal mucus organization could be observed.

**Figure 5:**
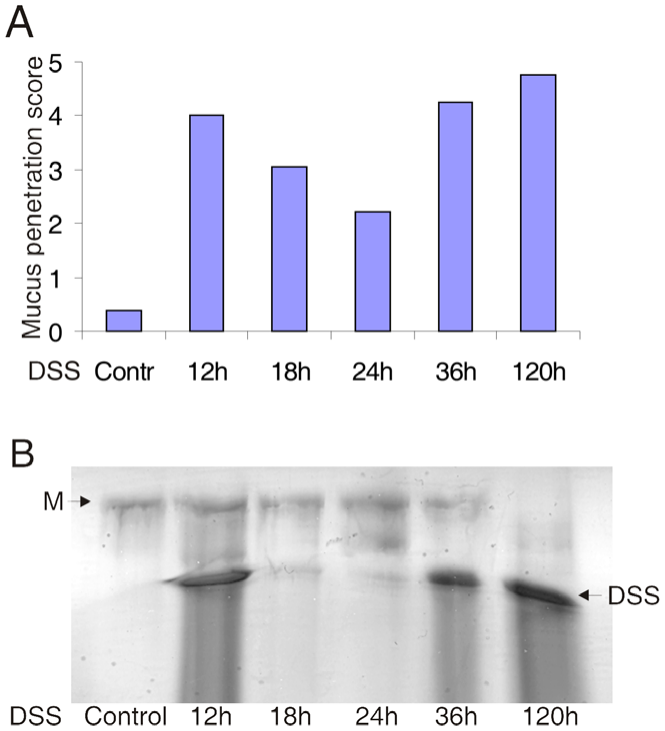
High number of bacteria penetrating the inner mucus layer co-varies with high amounts of DSS in the colon mucus. (A) Scoring of bacteria penetration of the inner firm mucus layer on a scale from 0 (no penetration) to 5 (large number of bacteria reaching the epithelial surface) of controls and DSS treated mice for indicated times. The scoring system is exemplified and demonstrated in Supplement Fig. S1. Mean values are for three mice (one mouse at 120 h) scored at four sites by two individuals in a blinded fashion. (B) Mucus from the analyzed animals was extracted with guanidinium chloride, the soluble fraction was reduced, separated on AgPAGE, and the gel stained with Alcian blue. The Muc2 monomer is marked by M. The DSS was also stained and is as expected absent in the untreated control animals. The amount of DSS reflects the animals diurnal activity and drinking rhythm as 12 and 36 h are after a night of activity and thus show high levels of DSS compared to 18 and 24 h that are after an inactive daytime that show low levels of DSS.

To further evaluate bacterial penetration of the inner mucus and the closeness of bacteria to the epithelial cells, a scoring system from 0–5 was used. Here 0 means no bacteria penetrating the inner mucus layer and 5 means a large number of bacteria in direct contact with the epithelium. This scoring system is explained and exemplified in Fig. S1. Using this system, blinded tissue sections were evaluated by two independent examiners and the average score is presented in Fig. 5A. A high score of 4 representing a large number of bacteria penetrating down to the epithelium, was reached already after 12 h. During the following 12 h there was a transient decline in the score value. This variation could be due to the murine diurnal rhythm of activity and drinking. To evaluate this possibility, the DSS concentration was measured in mucosal scrapings from mouse colon exposed to DSS. The scrapings were extracted in guanidinium chloride and analyzed by AgPAGE for large molecules (Fig. 5B). The guanidinium chloride insoluble Alcian blue stained Muc2 band showed no alterations (data not shown). The guanidinium chlorides soluble fraction contained the DSS that had accumulated in the distal colon in addition to some Muc2 (marked M). Fig. 5B show high amounts of DSS at 12 h, 36 h and 120 h, but low levels at 18 h and 24 h. Thus there is a direct co-variation between a high bacterial penetration score and high relative amounts of DSS in the mucus. This suggests a direct relation between the DSS amounts in the colon mucus and bacterial penetration of the inner mucus layer. These results further confirm that the DSS effect is fast and direct just as observed for the DSS treated mucus on the tissue explants. The *in vivo* experiments also suggest that the effect is reversible, at least at the standard early time points for evaluating DSS treatment.

### DSS and bacteria are in contact with the epithelium after 12 h of DSS exposure

**Figure 6:**
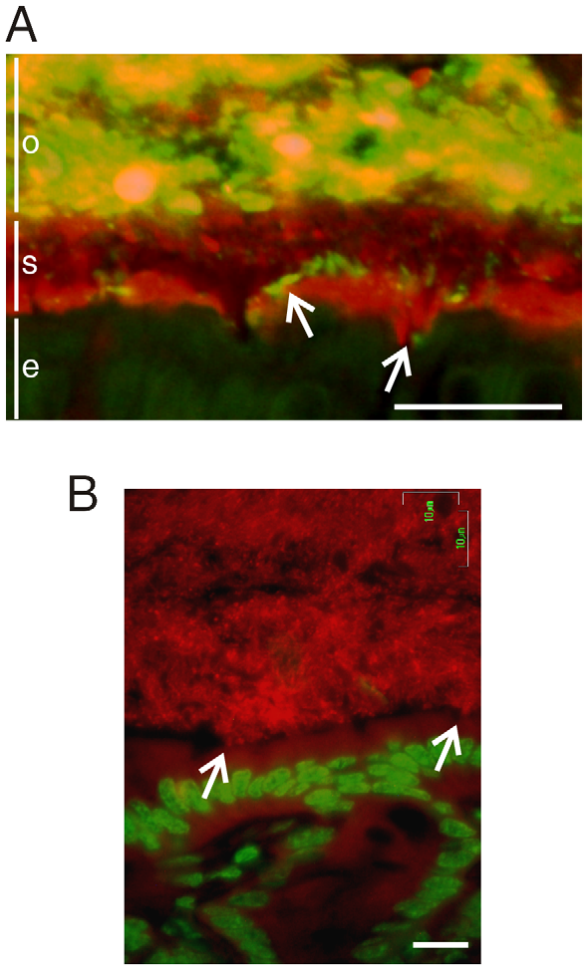
DSS treatment of mice allowed bacteria to penetrate into the firm mucus layer and come in direct contact with the epithelium. (A) Mice were given FITC-DSS (3%, green) in the drinking water for 12 h. The distal colon was fixed and the sections were immunostained with anti-Muc2 antiserum and anti-rabbit-Alexa546 (red). The arrows point at FITC-DSS that has penetrated the inner stratified mucus layer (*s*). Large amounts of bacteria and FITC-DSS is shown in the outer loose mucus layer (*o*). Epithelial cells are marked (*e*). Scale bar is 25 µm. (B) Bacteria were stained with FISH using the general rRNA probe EUB338 conjugated to Alexa 555 (red) and the nuclear DNA stained with Sytox Green DNA stain (green). The picture shows massive amounts of bacteria in contact with the epithelial cells. Scale bar is 10 µm.

In the explant system the DSS was able to diffuse into the firm mucus and when tested *in vivo* FITC labeled DSS was observed at the epithelial surface. The FITC conjugated DSS was given to the mice in the drinking water and already after 12 h a substantial amount had reached down to the epithelial cells (Fig. 6A). The penetration of bacteria into the mucus is thus simultaneous with DSS penetration into the mucus, suggesting that DSS alter the mucus properties in such a way that it allows bacteria to penetrate the otherwise impermable mucus. As shown in Fig. 6B an enormous bacterial load is observed on the epithelial surface already after 12 h DSS treatment. This massive bacterial contact can be envisioned as an explanation for the, until now unexplained, inflammation induced by orally administered sulfated Dextran molecules.

## Discussion

We could recently show that the inner mucus layer of colon is devoid of bacteria as it probably acts as a ‘filter’ that blocks the penetration of bacteria [2]. This explanation is supported by our observation here that fluorescent beads with a diameter in the same size range as bacteria (2 µm) do not penetrate the mucus formed on top of tissue explants. Using the most commonly used animal model for colitis, we have now shown that DSS in the drinking water allows bacteria to enter and penetrate the inner mucus layer before any inflammation can be observed. The earliest time point that we could observe bacteria penetrating the inner mucus layer was after 4 h, although the full effect was observed after 12 h. The observed correlation between the amount of DSS in the colon and the penetration of bacteria suggests that the effect is direct, fast, and initially reversible. That DSS can cause a fast alteration in the mucus permeability was also evident from the results showing that 2 µm fluorescent beads were able to penetrate the mucus plume formed on top of tissue explants after only 15 min. The fast DSS effect on already formed mucus was further illustrated by the observation that DSS significantly reduced the mucus thickness *in vitro* within 15 min.

As the DSS effect is fast and exerts its action on already formed mucus, it is unlikely that DSS alters the biosynthesis and formation of new mucus. Instead DSS seems to have a direct effect on the mucus biophysical structure. The reason for this effect on the main mucus component, the MUC2 mucin, is not understood. The glycans on the MUC2 mucin from human colon carries multiple negative charges on both sialic acid and sulfate residues [19]. It is thus likely that the highly sulfated DSS is readily soluble in the highly sulfated MUC2 mucin network. This was confirmed, but there was no major difference in the penetration of DSS and Dextran when FITC-labeled conjugates were tested, since both compounds reached the epithelial cells within 15 min. This means that the high amount of bound sulfate on DSS must destabilize the interactions that maintain the organization of the mucus. In fact, this is supported by the immunohistochemistry pictures showing that the typical stratified lamellar appearance of the inner mucus layer was lost after DSS exposure. Dextran, which does not contain the sulfate groups, does not affect the mucus organisation. The importance of mucin sulfation for mucus function is further illustrated by the observation that mice lacking the Nas1 sulfotransporter show less sulfation of their mucins and that these animals are more susceptible to colitis [20]. The sulfation is thus very important and colitis is observed, although less severe, using also DSS with a molecular mass of only 5 kDa [21]. Interestingly, DSS with a molecular mass of 500 kDa did not cause any inflammation [21]. The reason for this might be that it was too large to easily diffuse into the mucus network. DSS and Dextran with a molecular mass of 40–50 kDa, as normally used for the generation of DSS colitis can, however, penetrate the mucus quickly. DSS with all its sulfate groups are acidic in nature and one can speculate that the DSS effect on colon mucus could be similar to the pore forming effect of acid secreted from the stomach glands in the inner mucus layer covering the stomach epithelium [22]. The DSS sulfate groups might thus mimic the effect of hydrochloric acid in the stomach by opening pores in the colonic mucus.

The normal approach for the generation of DSS colitis in rodents is to use 3 to 5% of DSS for 5–7 days. The DSS passes along the gastrointestinal tract without being degraded and the water absorption in the colon will probably give a higher concentration than that given in the drinking water. We chose to use 3% for both our *in vivo* and *in-vitro* experiments. However, still we could record the dramatic effects on the mucus. In the DSS colitis model, an overt inflammation is observed after 3–5 days. The DSS effect observed here already after 12 h precedes inflammation and gives a relatively wide window during which the more typical colitis inflammation can develop. Studies of how the epithelial and immune systems are handling the bacteria during this time window should cast further light on the pathogenesis of colitis.

The DSS effects on the epithelium has been associated with increased permeability and disruption of tight junctions, however epithelial barrier dysfunction alone is not sufficient to cause disease [23]. Disrupted epithelial junction barrier leading to inflammation also results in adherent bacteria on the epithelial surfaces [24]. During the short times of DSS exposure investigated here we explored if there were any toxic effects on the epithelium. Tissue sections revealed normal histology at 12 and 24 h. The epithelial cells had a normal function after 24 h of oral DSS administration as the tissue was able to secrete and generate a mucus layer with normal thickness. This mucus was secreted without being exposed to DSS, further supporting that the initial effects of DSS is on the mucus itself. The Muc2 mucin builds the structure of the mucus as it is the major component and has the biochemical properties in its oligomerized form to form net-like structures [8]. Analysis of the Muc2 mucin by gel electrophoresis and proteomics did not reveal any difference after 24 h of DSS intake. Thus we conclude that the epithelial cells seem to be functional and that they secrete a normal mucus layer after 24 h or less of DSS treatment.

In mice with a thinner or no functional mucus layer, as for example germ-free mice, DSS treatment induces an acute and massive bleeding long before any inflammation is observed [20], [25], [26]. This effect is thus different from the one normally obtained by DSS treatment in wild-type mice showing a chronic inflammation that develops after several days. Why do then the germ-free mice react differently? One possible explanation is that these animals have a very thin inner mucus layer [2]. This might give a faster and more direct access of the DSS to the epithelial cells, thus exposing these for higher concentrations of DSS. This might be related to the toxic effects of DSS observed on cells in culture with 3% DSS in the culture media where the cells shrink together and do not survive. A possible reason for this direct DSS toxicity is that DSS is an effective calcium ion chelator.

Mice with different genetic backgrounds are different in their susceptibility to DSS treatment, but here we only used C57/Bl6 mice [27]. Relatively large differences in inflammatory scores are also observed when comparing different animal facilities [14], [15]. These differences are most likely due to variation in the bacterial flora. The animals in our facility show a relatively weak inflammation compared to others and the signs of inflammatory related alterations in the mucosa could not be observed at short DSS exposure times. That DSS generated colitis involves reactions to the enteric bacteria is suggested by studies showing that antimicrobial treatment using Cathelicidin ameliorated inflammation and colitis [28]. Although the reason for DSS causing colitis might be a direct toxic effect on the epithelium, it is more likely that this is due to the currently observed alterations in the inner mucus layer, and that DSS treatment allows bacteria to penetrate this inner mucus layer. Once the bacteria are allowed to penetrate the inner mucus layer, the situation will be similar to the one observed in mice lacking the Muc2 mucin [2]. These animals have a chronic inflammation with bloody diarrhea and will later develop colon cancer. A similar type of inflammation is also observed in two mouse strains with spontaneous mutations in the Muc2 mucin that does not allow the biosynthesis of a functional Muc2 mucin. These animals probably lack or have a defective inner mucus layer that is unable to protect the epithelium [29].

Our observation suggest that an intact inner mucus layer is instrumental for the protection of the colon epithelial cells and suggests that bacteria in contact with the epithelium will trigger the immune system to an inflammatory reaction. The cause of the inflammatory bowel disease UC is not understood and probably heterogeneous, but the importance of commensal bacteria is evident. It is also evident that a strong adaptive immune response is driving and maintaining the inflammation once started. Our observations of an inner mucus layer that is normally devoid of bacteria and that manipulations of this allow bacteria to reach to the epithelium suggest a new model for the pathophysiology of UC. As long as the massive amounts of bacteria are kept at a distance from the colonic epithelium and the immune system, the system is in balance. However, if bacteria come in contact with the epithelial cells, enter the crypts and are taken up by epithelial cells, the immune system will be triggered and start to react against also relatively harmless commensal bacteria. The inflammation may be detrimental to the normal homeostatic mechanisms of colon and also affect the inner mucus layer. Defects in the inner mucus layer is a possible new etiological mechanism behind UC.

## Methods

### Animals

All mice were inbred on the C57Bl/6 background from Taconic (Ejby, Denmark). Experimental animals were 10–12 weeks old males except for animals used for mucus permeability measurements that were 11–14 week old male mice. The mice were kept in individually ventilated cages under standardized conditions of temperature (21–22°C) and illumination (12 h light/12 h dark) under specific pathogen free conditions. They were given food (Labfor R34, Lantmännen, Stockholm, Sweden) and water ad libitum. Animal experimental procedures were approved by the Swedish Laboratory Animal Ethical Committee in Gothenburg was conducted in accordance with guidelines of the Swedish National Board for Laboratory Animals (#358-2009).

### Patients

Study subjects were recruited among patients referred to colonoscopy at Sahlgren's University Hospital, Gothenburg, Sweden. Six patients were included (Female, 28 years, rectal bleeding (haemorrhoids); Female, 44 years, abdominal pain.; Female, 44 years, anemia; Female, 69 years, control after diverticulitis; Male 84 years, rectal bleeding; Male 76 years, rectal bleeding (haemorrhoids)). Biopsies were taken in the sigmoid colon. All patients had a normal mucosa upon visual examination by the endoscopist and were regarded as normal controls. Written informed consent was obtained from the patients and approval was granted by the Human Research Ethical Committee of the Medical Faculty, University of Gothenburg, Gothenburg, Sweden (#040-08).

### Chemicals

Dextran Sodium Sulfate (DSS, average Mr of 48,800, 17.1% Sulfur substitution, 0.1% free Sulfate, pH 6.6), FITC-DSS (average Mr of 41,000, 1.6 mg FITC/g) and Dextran 20 was obtained from TdB Consultancy AB (Uppsala, Sweden). All other chemical were from Sigma-Aldrich (St. Louis, MO).

### Measurement of mucus thickness in vitro on explant cultures of mouse and human colon

*In vitro* measurement of mucus alterations by DSS were done as described here. Mice were anaesthetized with 3.7% isoflurane and euthanized by cervical dislocation. The distal colon was dissected and flushed with oxygenated ice-cold KREB [115.8 mM NaCl, 1.3 mM CaCl_2_, 3.6 mM KCl, 1.4 mM KH_2_PO_4_, 23.1 mM NaHCO_3_ and 1.2 mM MgSO_4_]. The specimen was then opened along the mesenteric border, stripped of the muscle layer and mounted in the RC-50 image chamber (exposed area 1.77 mm^2^) (Warner instruments, Hamden, CT). The basolateral side of the chamber was constantly perfused (6 ml/h) with oxygenated KREB solution with supplements [5.7 mM Na-Pyruvate, 5.13 mM Na-L-Glutamate and 10 mM D-Glucose, pH 7.4]. The apical side of the chamber was bathed in 100 µl oxygenated KREB solution with supplents [5.7 mM Na-Pyruvate, 5.1 mM Na-L-Glutamate and 10 mM D-Mannitol, pH 7.4]. The temperature was kept at 37°C throughout the whole experiment.

Biopsies obtained from sigmoid colon were instantly put into ice-cold oxygenated KREB solution and kept on ice until mounting in the RC-50 imaging chamber. The following procedure was identical to the one described for mouse tissue.

The thickness of the mucus layer was assessed by measuring the distance between the epithelial surface and the surface of the mucus layer using a micropipette (Sutter instruments, CA) connected to a micropuller (55° angle) (in-house made) and observed through a stereomicroscope (Leica, Wetzlar, Germany). Digital recording of the measurements was enabled by connecting the micropuller to a digimatic indicator (Mitutoyo, Kawasaki, Japan). To visualize the surface of the mucus layer a suspension of activated charcoal was added. The thickness was measured with 15 min intervals for a total time of 60 min. During each measuring event five recordings were made and the calculated mean value was used as a single measurement. The vertical distance between the epithelial surface and the mucus surface was calculated by multiplying the obtained value with cosin55°. At time 45 min the loose mucus layer was removed by suction and the thickness of the firmly adherent mucus layer was measured. To assess the effect of DSS and Dextran on the thickness of the mucus layer the apical solution was replaced with KREB mannitol solution containing either 3% DSS or 3% Dextran and incubated for 15 min. After 15 min of incubation the loose mucus layer was removed by suction and the thickness of the firmly adherent layer was measured. Data is presented as mean ± SEM. Effects of the treatments were analyzed by using the student's t-test. A p-value<0.05 was considered as statistically significant.

### Confocal microscopy of the effects of DSS on mucus properties

The effects of DSS on mucus permeability were studied using the RC-50 imaging chamber mounted onto an upright confocal microscope. The protocol used for isolating the tissue, running the chamber and the solutions used in these experiments were the same as described for the mucus thickness measurements. The colonic epithelium was labeled using CellTracer BODIPY TR methyl ester (Invitrogen, Carlsbad, CA) added to the KREB solution and the basolateral perfusate (2 µl/ml). An additional incubation of 20 min in KREB-Bodipy solution was added after removing the muscle layer. After 45 min incubation in the RC-50 imaging chamber yellow-green fluorescent beads (2 µm FluoSpheres, Invitogen, Carlsbad. CA) were added to the apical surface and allowed to sediment down to the mucus layer. Confocal images were taken in a XY stack with an optical section of 13.6 µm in 3 µm intervals using a BioRad Radiance 2000 imaging system and a 10× objective. To assess the effects of DSS on mucus permeability the apical solution was replaced by KREB solution containing either 3% DSS or 3% Dextran as described above. A second XY stack was taken after 15 min of incubation in DSS or Dextran. Images were processed using the Laser sharp 2000 software and Image J. The Z-axis section was used to present the results.

### DSS treatment of mice

3% DSS or FITC-DSS was administered orally in the drinking water for 12 to 120 h starting at 8.00 p.m. (dark) to assure activity of the animals at the start of the experiment. The DSS had a molecular mass of 49 kDa and had 17% sulfate substitution. The FITC-labeled DSS had 1.6 mg FITC/g DSS. Each experimental time point included 3 animals except for the 120 h control where one animal was used.

### In vivo mucus measurements after DSS treatment

*In vivo* measurements of the firm mucus were performed as described previously on animals subjected to 3% DSS in the drinking water for 24 h (n = 3) or controls (n = 6) [2], [30]. During the 1 h stabilization period spontaneous mucus secretion occurs producing the full mucus layers in the measurement chamber. The secreted mucus is not subjected to additional DSS.

### Preparation and extraction of mucus

Loose and firm mucus from the *in vivo* measurements was collected by suctioning (loose) or gentle scraping (firm) in PBS supplemented with Complete EDTA-free protease inhibitor (Roche, Basel, Switzerland) and the samples were frozen.

Total mucus from the distal colon of DSS treated animals was removed by gentle scraping in PBS supplemented with Complete EDTA-free protease inhibitor (Roche, Basel, Switzerland). The mucus samples were extracted three times in guanidinium chloride [6.0 M GuHCl, 5 mM EDTA, 0.1 M Tris-HCl, pH 8.0] by rotation at +4°C over night and centrifuged for 20 min at 16,000×g. The resulting soluble and insoluble fractions were separated and dialyzed against water. The soluble fraction contained the luminal content including the DSS.

All samples were incubated with sample buffer [0.75 M Tris-HCl pH 8.0, 2% SDS, 0.01% Bromophenol blue, 60% glycerol, 100 mM DTT] at 95°C for 10 min with continued reduction at 37°C for 2 h.

### SDS-agarose composite gel electrophoresis for separation of mucins

The reduced samples were analyzed by composite agarose-polyacrylamide gel electrophoresis (AgPAGE) with a gel containing agarose (0.5–1% gradient), acrylamide (0–6%) and glycerol (0–10%) [31]. The electrophoresis was performed on ice at +4°C for 16 h at 12 mA/gel. The gel was stained with Alcian blue visualizing both glycosylated mucins and DSS.

### Histology and Immunostaining

Segments of the distal colon from mice were fixed in water-free Methanol-Carnoy's fixative [60% dry methanol, 30% chloroform and 10% acetic acid]. The tissue was washed in methanol before embedded in paraffin and sectioned, 4 µm. The sections were dewaxed using Xylene substitute (Sigma, St. Louis, MO) and hydrated. The antigens were retrieved by microwave heating in 0.01 M citric buffer pH 6 and the sections were stained with Haemtoxilin/Eosin or by the anti-MUC2C3 antiserum [2]. FITC conjugated goat anti-rabbit immunoglobulins (DAKO, Copenhagen, Denmark) or Alexa 546 conjugated goat anti-rabbit immunoglobulins (Invitrogen, Carlsbad, CA) were used as secondary antibodies and DNA was stained by DAPI or Sytox Green DNA stain (Invitrogen, Carlsbad, CA). Pictures were obtained using an Eclipse E1000 (Nikon, Tokyo, Japan) fluorescence microscope.

### Fluorescent in situ hybridization

Paraffin sections were dewaxed with Xylene substitute and hybridized with a general bacterial probe, EUB 338 conjugated to Alexa 555 as described previously [2]. Immunostaining after hybridizations was performed at +4°C without antigen retrieval. Pictures were obtained in an Eclipse E1000 (Nikon, Tokyo, Japan) fluorescence microscope.

### Bacterial penetration score

The bacterial penetration score system was worked out from the sections. 0 representing complete separation of bacteria and epithelium by mucus and 5 indicates close contact between the epithelium and a large amount of microbes. Pictures representative of the different scores are presented in Fig. S1. Colon sections from 3 animals were stained with the Muc2-C3 antiserum and DAPI DNA staining. Four separate areas from each animal were scored by two individuals independently in a blinded fashion. Mean values of the scores are presented.

## Supporting Information

Figure S1 Scoring system for the evaluation of bacterial penetration of the inner mucus layer. Sections of colon from the DSS treated animals and controls were stained for Muc2 (green, left panels) and DAPI (blue, middle panel) to visualize DNA including bacteria. The right panel shows the merge. The scoring of bacterial penetration of the mucus was from 0 to 5. No bacteria into the inner mucus layer were set to score 0 and massive contact between bacteria and epithelium was set to 5. Intermediate scores were set according to the pictures.
(0.91 MB PDF)
