# Supplementary material for: Bacteria Penetrate the Inner Mucus Layer before Inflammation in the Dextran Sulfate Colitis Model
Source: PLoS One. 2010 Aug 18;5(8):e12238. doi: 10.1371/journal.pone.0012238 (PMC2923597; doi:10.1371/journal.pone.0012238)
Supplement: Figure S1 — Scoring system for the evaluation of bacterial penetration of the inner mucus layer. Sections of colon from the DSS treated animals and controls were stained for Muc2 (green, left panels) and DAPI (blue, middle panel) to visualize DNA including bacteria. The right panel shows the merge. The scoring of bacterial penetration of the mucus was from 0 to 5. No bacteria into the inner mucus layer were set to score 0 and massive contact between bacteria and epithelium was set to 5. Intermediate scores were set according to the pictures. (0.91 MB PDF) [file pone.0012238.s001.pdf]

SUPPLEMENTARY FIGURE TO

Bacteria penetrate the inner mucus layer before inflammation in the Dextran sulfate colitis model

Malin E. V. Johansson, Jenny K. Gustafsson, Karolina E. Sjöberg, Joel Pettersson, Lena Holm, Henrik Sjövall, and Gunnar C. Hansson

Figure S1. Scoring system for the evaluation of bacterial penetration of the inner mucus layer. Sections of colon from the DSS treated animals and controls were stained for Muc2 (green, left panels) and DAPI (blue, middle panel) to visualize DNA including bacteria. The right panel shows the merge. The scoring of bacterial penetration of the mucus was from 0 to 5. No bacteria into the inner mucus layer were set to score 0 and massive contact between bacteria and epithelium was set to 5. Intermediate scores were set according to the pictures.

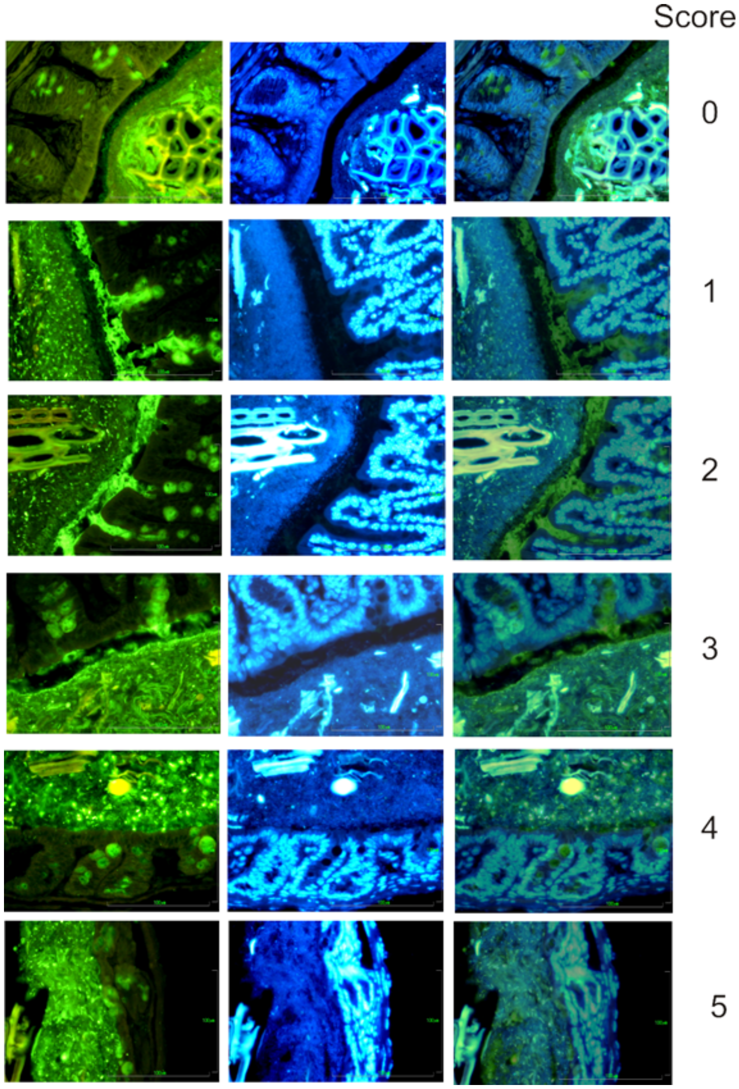
Score
0
1
2
3
4
5
